# Can a Rule-Based Expert System Diagnose Nasal Obstruction from Nasoendoscopy Videos?

**DOI:** 10.1055/a-2656-6413

**Published:** 2025-07-28

**Authors:** Annakan Navaratnam, Nonpawith Phoommanee, Vikas Acharya, Alfonso Luca Pendolino, Terence S. Leung, Peter J. Andrews

**Affiliations:** 1 Department of ENT, Royal National ENT and Eastman Dental Hospitals, London, United Kingdom; 2 Department of Medical Physics and Biomedical Engineering, University College London, London, United Kingdom; 3 UCL Ear Institute, University College London, London, United Kingdom; 4 Department of ENT, Imperial College Healthcare NHS Trust, London, United Kingdom

**Keywords:** nasal obstruction, nasal endoscopy, rule-based expert systems

## Abstract

**Introduction:**

Nasal obstruction has multiple causes requiring specialist endoscopy for diagnosis. A rule-based expert system (RB-ES), which applies five “if–then” rules based on nasal features, may help replicate ENT decision-making in settings with limited access.

**Objectives & Hypotheses:**

This study evaluated RB-ES in diagnosing allergic rhinitis, chronic rhinosinusitis with (CRSwNP) and without (CRSsNP) nasal polyps, and deviated nasal septum. Primary outcomes were sensitivity and specificity; the secondary outcome was agreement with ENT specialists.

**Study Design:**

Prospective cohort study.

**Methods:**

Seventy-one participants (65 patients, 6 controls) underwent pre- and postdecongestion endoscopy. Four ENT specialists provided diagnoses. RB-ES performance was compared against confirmed clinical diagnoses.

**Results:**

RB-ES showed no detectable significant sensitivity differences from ENT specialists (all *p* > 0.05). Sensitivity was highest for CRSwNP; specificity remained high overall.

**Conclusion:**

RB-ES matched specialist performance in CRSwNP diagnosis. Dataset expansion and artificial intelligence integration are recommended for further validation.

**Level of Evidence:**

II.

Allergic rhinitis (AR), chronic rhinosinusitis with (CRSwNP) or without (CRSsNP) nasal polyps, and a deviated nasal septum (DNS) are the most common causes of nasal obstruction in ENT patients. In the United Kingdom, AR and CRS affect over 20% and 10% of the population, respectively. 1 2 3 Diagnosing nasal obstruction is complex due to overlapping structural (DNS) and inflammatory (AR, CRSwNP, CRSsNP) causes. A definitive diagnosis requires nasal endoscopy alongside allergy tests and CT scans, 4 while blood tests are not routinely used. 5 6

In the United Kingdom's primary care, diagnosing nasal obstruction is challenging due to the absence of allergy testing, CT scanning, and nasal endoscopy. Diagnosis is often based on clinical history and basic anterior nasal examination with a pen torch, 7 leading to reliance on ENT specialists for further assessment. A standardized minimum dataset of nasal endoscopic findings for common nasal obstruction causes is lacking.

Research has focused on key anatomical structures in nasal obstruction assessment, using anterior rhinoscopy or nasal endoscopy. 8 While nasal polyp 9 10 and DNS classifications are well studied, internal nasal valve (INV) competency 11 and inferior turbinate (IT) size 12 have been less extensively assessed. 13 The minimum classification criteria for diagnosing nasal obstruction via endoscopy remain undefined, limiting potential artificial intelligence (AI) automation.

Expert systems analyze clinical data to assist in disease diagnosis and improve decision-making. These systems typically include long-term and short-term memory, an inference engine, and sometimes an explanation module. 14 Rule-based expert systems (RB-ES) support health care professionals in diagnostics, decision-making, and as alternatives when specialists are unavailable.

Machine learning (ML) techniques, including neural networks, fuzzy logic, and support vector machines, address complex medical challenges. According to Freitas, knowledge representation falls into five types: Decision tables, classification rules, decision trees, Bayesian networks, and nearest neighbor algorithms. 15 Clinical decision-making in nasal obstruction assessment aligns with decision table processes, making them a suitable knowledge representation structure.

Nasal pathology imposes a significant burden on the United Kingdom's general practitioners, where consultations are time-limited and access to rhinology diagnostics is restricted. This often results in unnecessary and costly referrals to secondary and tertiary ENT centers. Streamlining patient pathways through community-based diagnostics could reduce referrals. RB-ES, enhanced by AI applications, could help address this need.

This study evaluates the sensitivity and specificity of a decision table-based RB-ES in diagnosing nasal obstruction causes from nasoendoscopy videos of the anterior nasal cavity using established classification systems.

## Materials and Methods

### Data Collection

The study received full ethical approval from the London – City & East Research Ethics Committee (reference: 15/LO/0187), and all participants provided written informed consent. We recruited 71 participants, including 65 patients and 6 controls (age ≥18) with no history of rhinological conditions. Each participant underwent a comprehensive clinical evaluation, including nasoendoscopy, medical history, questionnaires, skin prick test, and a CT scan of the sinuses. An ENT specialist reviewed anterior nasal cavity videos (comparable to anterior rhinoscopy) and integrated all test results to confirm diagnoses of AR, CRSsNP, CRSwNP, or DNS.

Nasal cavity recordings were captured using a Video Naso-Pharyngo-Laryngoscope (VNL9-CP; Pentax Medical) and processed with the VIVIDEO Video Processor (CP-1000) (Pentax Medical). A nasal decongestant spray (two puffs of xylometazoline hydrochloride 0.1% w/v per nostril) was applied, and recordings were repeated 10 minutes later to assess endoscopic changes.

### Video Review and Decision Table Formulation

Participant order was randomized, and ENT specialists were blinded to patient data. Participants were divided into seven Microsoft Forms questionnaire sets (9–11 participants per set), each containing pre- and postdecongestant videos. Reviews were designed for completion within 1 hour per set and conducted in compliance with the General Data Protection Regulation (GDPR).

### Video Review and Grading

The grading process utilized validated classification systems to evaluate nasal conditions systematically. 11 12 16 17 As summarized in Table 1 , these systems were selected for their clinical relevance and ease of application in diagnosing conditions such as DNS, CRSwNP, AR, and CRSsNP. The classifications provided standardized grading criteria and helped differentiate conditions based on responses to nasal decongestants. To enhance accuracy, the INV grading 11 was modified to allow ENT specialists to assign certainty levels for Grades 0 and 1, introducing intermediate categories of “maybe 0” (1/3) and “maybe 1” (2/3), while the other classifications remained unchanged. 12 16 17

**Table 1:** Selected studies on nasal obstruction classification systems

Anatomical structure	Grading description				
	Grade 0	Grade 1	Grade 2	Grade 3	Grade 4
Internal nasal valve 11	The head of the MT can be seen clearly	The MT is partially blocked from the view	The MT cannot be seen at all	N/A	N/A
Inferior turbinate 12	N/A	Up to 25% of the airway space was occupied	Up to 50% of the airway space was occupied	Up to 75% of the airway space was occupied	Up to 100% of the airway space was occupied
Deviated nasal septum 16	Septum straight	Deviated nasal septum covers up to one-third of the nasal cavity	Deviated nasal septum covers up to two-thirds of the nasal cavity	Deviated nasal septum covers above two-thirds of nasal cavity	N/A
Nasal polyp 17	No nasal polyps visualized	Small nasal polyps visible in middle meatus	Middle meatus completely filled with nasal polyps	Nasal polyps extending out of middle meatus but above the IT	Nasal polyps completely fill entire nasal cavity

Abbreviation: IT, inferior turbinate; MT, middle turbinate.

N/A indicates that grading is not applicable.

During the review, each ENT specialist graded the videos according to these classification systems and provided a predicted diagnosis for each participant. Specialists categorized participants as either controls or as having at least one of the following conditions: AR, CRSwNP, CRSsNP, or DNS. This structured evaluation facilitated a comprehensive and standardized approach to nasal condition assessment.

### Rule-Based Expert System and Decision Table Formulation

**Fig. 1:**
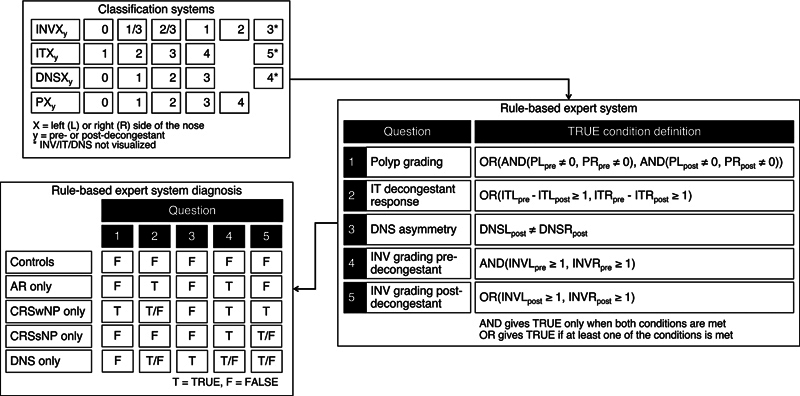
Rule-based expert system employing the nasal obstruction classification systems as inputs, where X denotes either the left (L) or right (R) nasal cavity, and y signifies either the pre- or postdecongestant status. The diagram illustrates a predicted diagnosis, wherein the patient only has a single condition of nasal obstruction. The full predicted diagnosis, encompassing scenarios where the patient presents with multiple conditions, can be found in Table 2 . AR, allergic rhinitis; CRSsNP, chronic rhinosinusitis without nasal polyps; CRSwNP, chronic rhinosinusitis with nasal polyps; DNS, deviated nasal septum; INV, internal nasal valve; IT, inferior turbinate.

The RB-ES was developed based on the consensus of four ENT specialists to prioritize clinical features for diagnosis. It classifies participants using binary outcomes for five diagnostic questions (see Fig. 1 ).

#### Step 1: Nasal Polyps Detection (Question 1)

RB-ES first assesses nasal polyps, a key criterion for CRSwNP diagnosis. Bilateral nasal polyps confirm CRSwNP, while unilateral nasal polyps may indicate other pathologies, including tumors, which were excluded from this study. Although RB-ES could be adapted for unilateral polypoid findings, differentiating inflammatory from neoplastic lesions requires imaging or histopathology. Studies support polyp detection via both subjective assessment and ML. 18 19 20 21 22 23 24 25

#### Step 2: Decongestant Response (Question 2)

Bilateral IT grading differentiates AR from CRSsNP based on nasal decongestant response. A ≥35% IT size reduction on both sides suggests AR, as allergic inflammation causes reversible congestion. 26 CRSsNP, a chronic condition, typically does not show this response. This threshold aligns with Ciprandi et al., 26 who found that non-AR patients exhibit <35% airflow change after decongestant use. A unilateral reduction may be normal or indicative of a control patient.

#### Step 3: Deviated Nasal Septum Asymmetry (Question 3)

DNS is identified by evaluating asymmetry in DNS grading on both nasal cavities postdecongestant. This approach minimizes the influence of the nasal cycle, ensuring more accurate classification.

#### Steps 4 and 5: Internal Nasal Valve Grading (Pre- and Postdecongestant)

INV grading differentiates controls from other diagnoses. Controls are graded below 1 predecongestant on both sides (i.e., both MTs clearly visible: grade 0, maybe 0, or maybe 1) and maintain this postdecongestant. A grade of 1 or higher on both sides of pre- and postdecongestant suggests pathology. INV grading correlates with unilateral peak nasal inspiratory flow (PNIF) and visual analog scale (VAS) scores 11 and guides in the diagnosis of controls. CRSsNP returns TRUE for Question 4 due to persistent mucosal obstruction, but often returns FALSE postdecongestant (Question 5).

RB-ES generates TRUE/FALSE outcomes based on input parameters (INV, IT, DNS, polyp grading). Expected results for different groups are as follows:

- **Control** : FALSE for all questions.
- **AR-only** : TRUE for Questions 2 and 4 and FALSE for the remaining questions.
- **CRSwNP-only** : TRUE for Question 1 and the remaining questions disregarded.
- **CRSsNP-only** : TRUE for Question 4 and FALSE for the remaining questions.
- **DNS-only** : TRUE for Question 3 (Questions 2, 4, and 5 are disregarded).

For overlapping conditions, specific rule permutations were prioritized based on expert consensus (see Table 2 ).

**Table 2:** The full decision table with combinations of conditions of nasal obstruction

Question					Conditions
1	2	3	4	5
T	T	T	T	T	AR + CRSwNP + DNS
T	T	T	T	F	AR + CRSwNP + DNS
T	T	T	F	T	CRSwNP + DNS
T	T	T	F	F	CRSwNP + DNS
T	T	F	T	T	AR + CRSwNP
T	T	F	T	F	AR + CRSwNP
T	T	F	F	T	CRSwNP
T	T	F	F	F	CRSwNP
T	F	T	T	T	CRSwNP + DNS
T	F	T	T	F	CRSwNP + DNS
T	F	T	F	T	CRSwNP + DNS
T	F	T	F	F	CRSwNP + DNS
T	F	F	T	T	CRSwNP
T	F	F	T	F	CRSwNP
T	F	F	F	T	CRSwNP
T	F	F	F	F	CRSwNP
F	T	T	T	T	AR + CRSsNP + DNS
F	T	T	T	F	AR + DNS
F	T	T	F	T	DNS
F	T	T	F	F	DNS
F	T	F	T	T	AR + CRSsNP
F	T	F	T	F	AR
F	T	F	F	T	Controls
F	T	F	F	F	Controls
F	F	T	T	T	CRSsNP + DNS
F	F	T	T	F	CRSsNP + DNS
F	F	T	F	T	DNS
F	F	T	F	F	DNS
F	F	F	T	T	CRSsNP
F	F	F	T	F	CRSsNP
F	F	F	F	T	Controls
F	F	F	F	F	Controls

Abbreviations: AR, allergic rhinitis; CRSsNP, chronic rhinosinusitis without nasal polyps; CRSwNP, chronic rhinosinusitis with nasal polyps; DNS, deviated nasal septum.

RB-ES is well-suited for automation, with AI-powered grading systems providing inputs (INV, IT, DNS, nasal polyp grading). Automating this process enhances consistency, reduces manual effort, and aligns with AI-driven health care innovations, ensuring precision and scalability in clinical use.

### Workflow

**Fig. 2:**
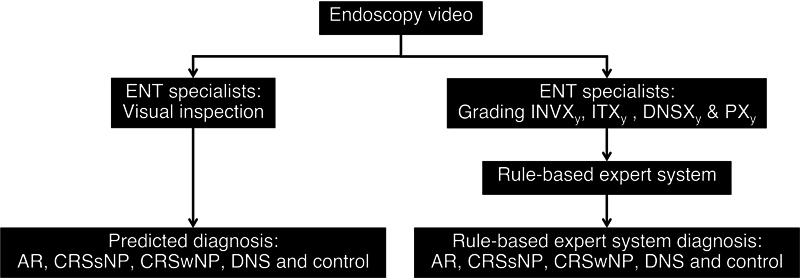
Workflow: ( **A** ) Predicted diagnosis obtained via visual inspection, and ( **B** ) rule-based expert system diagnosis using grading and RB-ES. AR, allergic rhinitis; CRSsNP, chronic rhinosinusitis without nasal polyps; CRSwNP, chronic rhinosinusitis with nasal polyps; DNS, deviated nasal septum; INV, internal nasal valve; IT, inferior turbinate; RB-ES, rule-based expert system.

The workflow ( Fig. 2 ) involved ENT specialists reviewing endoscopy videos to provide predicted diagnoses via visual inspection. Simultaneously, they graded four nasal obstruction classification systems, and these grades were input into the RB-ES ( Fig. 1 ) to generate diagnoses.

### Data Analysis

For specialist-specific results, each ENT specialist's assigned grades were directly input into the RB-ES for comparison with diagnoses from visual inspection. For overall results, final grades were determined by the mode among specialists who did not assign a visualized result. If no mode was available, the average was used, considering only valid grades. When all specialists assigned not visualized, predefined values ( Fig. 1 ) were used as RB-ES inputs. A diagnosis was considered positive if at least two specialists independently identified the condition. Sensitivity and specificity, with 95% confidence intervals (CIs), were calculated for both methods against the gold standard diagnosis (derived from medical tests and patient history) using the testCompareR package in R. 27

Conditional logistic regression assessed differences in sensitivity and specificity between methods. Power analysis estimated the minimum sample size needed for a meaningful difference at a 0.05 significance level (two-sided) and 80% power, assuming an effect size of 0.3 (moderate difference). With 71 participants, the study fell below this threshold, warranting cautious interpretation. All analyses, including regression and power calculations, were conducted in Python 3.11.

Interrater agreement among ENT specialists was assessed using Krippendorff's α, 28 calculated with the Κ-Alpha Calculator web application. 29 Agreement levels were defined as poor (κ ≤ 0.667), moderate (0.667 ≤ κ < 0.800), satisfactory (0.800 ≤ κ < 1.000), and perfect (κ = 1.000). 28

## Results

### Patient Demographics

The Nasal Endoscopy — University College London Hospitals (NE-UCLH) dataset includes 40 males and 31 females, with a mean age of 42.1 years (95% CI: 38.5–45.7). Table 3 presents participant demographics for controls and participants with nasal conditions (AR, CRSsNP, CRSwNP, DNS), illustrating condition distribution while accounting for overlap. Specific condition combinations are included for reference, but only controls and condition-inclusive groups are analyzed in subsequent sections.

**Table 3:** The table presents the number of participants categorized into controls, condition-inclusive cases, and specific condition combinations

Group	*n* (percentage)
Controls	6 (8.5%)
Condition inclusive	65 (91.5%)
Includes AR	32 (45.1%)
Includes CRSsNP	9 (12.7%)
Includes CRSwNP	14 (19.7%)
Includes DNS	35 (49.3%)
Specific conditions	
AR only	11 (15.5%)
CRSsNP only	5 (7.0%)
CRSwNP only	9 (12.7%)
DNS only	16 (22.5%)
AR + CRSsNP	2 (2.8%)
AR + CRSwNP	3 (4.2%)
AR + DNS	15 (21.1%)
CRSsNP + DNS	1 (1.4%)
CRSwNP + DNS	2 (2.8%)
AR + CRSsNP + DNS	1 (1.4%)
AR + CRSwNP + DNS	0 (0.0%)
Total	71 (100.0%)

The conditions are defined as follows: AR (allergic rhinitis), CRSsNP (chronic rhinosinusitis without nasal polyps), CRSwNP (chronic rhinosinusitis with nasal polyps), and DNS (deviated nasal septum).

### Comparing Diagnostic Performance between Visual Inspection and Rule-Based Expert System

Table 4 shows that both visual inspection and RB-ES exhibit varying sensitivity across conditions. For AR, CRSsNP, and controls, RB-ES sensitivities were 46.9% (95% CI: 30.8–63.5%), 55.6% (95% CI: 26.8–81.2%), and 50.0% (95% CI: 15.6–74.8%), all below the 65% threshold, indicating that nasoendoscopy videos alone are insufficient for diagnosis. CRSwNP had the highest sensitivities: 78.6% (95% CI: 52.9–92.9%) for visual inspection and 71.4% (95% CI: 45.7–88.6%) for RB-ES, suggesting viability for diagnosing this condition. DNS was borderline, with visual inspection reaching 65.7% (95% CI: 49.3–79.3%) and RB-ES reaching 60.0% (43.6–74.5%). Specificity was generally high, though it primarily indicates the ability to exclude non-cases rather than confirm diagnoses, reinforcing that neither method alone is sufficient.

**Table 4:** Comparison of sensitivity and specificity (percentage) between the visual inspection and rule-based expert system methods, both overall and for each ENT specialist

Condition	ENT specialists	Sensitivity (%)		Specificity (%)	
		Visual inspection	RB-ES	Visual inspection	RB-ES
AR	Overall	34.4 (20.3–51.6)	46.9 (30.8–63.5)	64.1 (48.5–77.3)	53.8 (38.6–68.5)
	Consultant 1	18.8 (8.6–35.1)	53.1 (36.5–69.2)	64.1 (48.5–77.3)	43.6 (29.3–59.0)
	Consultant 2	28.1 (15.4–45.2)	9.4 (2.9–23.9)	**71.8 (56.4** – **83.6)**	**71.8 (56.4** – **83.6)**
	Consultant 3	53.1 (36.5–69.2)	43.8 (28.1–60.6)	**69.2 (53.7** – **81.6)**	**69.2 (53.7** – **81.6)**
	Consultant 4	25.0 (13.1–41.9)	6.2 (1.4–19.8)	71.8 (56.4–83.6)	**84.6 (70.5** – **93.0)**
CRSsNP	Overall	44.4 (18.8–73.2)	55.6 (26.8–81.2)	**93.5 (84.7** – **97.6)**	53.2 (41.0–65.1)
	Consultant 1	44.4 (18.8–73.2)	55.6 (26.8–81.2)	**88.7 (78.6** – **94.6)**	45.2 (33.4–57.4)
	Consultant 2	22.2 (5.7–54.1)	0.0 (0.0–28.8)	**82.3 (71.1** – **89.9)**	77.4 (65.7–86.2)
	Consultant 3	44.4 (18.8–73.2)	55.6 (26.8–81.2)	**90.3 (80.6** – **95.7)**	58.1 (45.7–69.6)
	Consultant 4	44.4 (18.8–73.2)	0.0 (0.0–28.8)	**90.3 (80.6** – **95.7)**	88.7 (78.6–94.6)
CRSwNP	Overall	**78.6 (52.9** – **92.9)**	71.4 (45.7–88.6)	**96.5 (88.3** – **99.2)**	94.7 (85.8–98.4)
	Consultant 1	**85.7 (60.6** – **96.5)**	71.4 (45.7–88.6)	93.0 (83.5–97.4)	**94.7 (85.8** – **98.4)**
	Consultant 2	64.3 (39.0–83.9)	57.1 (32.7–78.7)	**96.5 (88.3** – **99.2)**	**96.5 (88.3** – **99.2)**
	Consultant 3	**78.6 (52.9** – **92.9)**	71.4 (45.7–88.6)	**94.7 (85.8** – **98.4)**	**94.7 (85.8** – **98.4)**
	Consultant 4	**78.6 (52.9** – **92.9)**	50.0 (26.8–73.2)	**96.5 (88.3** – **99.2)**	**96.5 (88.3** – **99.2)**
DNS	Overall	**65.7 (49.3** – **79.3)**	60.0 (43.6–74.5)	61.1 (44.9–75.3)	**77.8 (62.1** – **88.5)**
	Consultant 1	62.9 (46.4–76.9)	60.0 (43.6–74.5)	**77.8 (62.1** – **88.5)**	**77.8 (62.1** – **88.5)**
	Consultant 2	62.9 (46.4–76.9)	**65.7 (49.3** – **79.3)**	**72.2 (56.2** – **84.3)**	22.2 (11.5–37.9)
	Consultant 3	54.3 (38.2–69.6)	57.1 (40.9–72.1)	**75.0 (59.1** – **86.4)**	52.8 (37.0–68.0)
	Consultant 4	**80.0 (64.3** – **90.2)**	**80.0 (64.3** – **90.2)**	**52.8 (37.0** – **68.0)**	33.3 (20.1–49.5)
Controls	Overall	33.3 (9.2–69.5)	50.0 (18.8–81.2)	**83.1 (72.3** – **90.4)**	**83.1 (72.3** – **90.4)**
	Consultant 1	50.0 (18.8–81.2)	33.3 (9.2–69.5)	**86.2 (75.9** – **92.7)**	**86.2 (75.9** – **92.7)**
	Consultant 2	33.3 (9.2–69.5)	16.7 (2.1–55.4)	81.5 (70.6–89.2)	**89.2 (79.6** – **94.8)**
	Consultant 3	16.7 (2.1–55.4)	33.3 (9.2–69.5)	**83.1 (72.3** – **90.4)**	78.5 (67.1–86.8)
	Consultant 4	33.3 (9.2–69.5)	50.0 (18.8–81.2)	**84.6 (74.1** – **91.6)**	80.0 (68.9–88.0)

Abbreviations: AR, allergic rhinitis; CRSsNP, chronic rhinosinusitis without nasal polyps; CRSwNP, chronic rhinosinusitis with nasal polyps; DNS, deviated nasal septum; INV, internal nasal valve; IT, inferior turbinate; RB-ES, rule-based expert system.

Values are presented with 95% confidence intervals in parentheses. For each metric, the superior results between the two methods are highlighted in bold, provided they exceed 65%.

Table 5 compares visual inspection and RB-ES using conditional logistic regression. No significant sensitivity differences were found for AR ( *p* = 0.18), CRSsNP ( *p* = 0.57), CRSwNP ( *p* = 0.91), DNS ( *p* = 0.53), or controls ( *p* = 0.57), indicating RB-ES was as good as visual inspection by ENT specialists. Overall specificity advantage was not observed (all *p* > 0.05) when comparing RB-ES and visual inspection.

**Table 5:** *p* -Values from conditional logistic regression comparing the diagnostic performance of visual inspection and rule-based expert system methods, both overall and for each ENT specialist

Condition	ENT specialists	Sensitivity		Specificity	
		*p* -Value	Better method	*p* -Value	Better method
AR	Overall	0.18	NDD	0.26	NDD
	Consultant 1	0.02	Rules	0.046	Visual
	Consultant 2	0.08	NDD	>0.99	NDD
	Consultant 3	0.27	NDD	>0.99	NDD
	Consultant 4	0.08	NDD	0.18	NDD
CRSsNP	Overall	0.57	NDD	0.76	NDD
	Consultant 1	0.57	NDD	<0.001	Visual
	Consultant 2	0.92	NDD	0.51	NDD
	Consultant 3	0.57	NDD	0.001	Visual
	Consultant 4	0.91	NDD	0.74	NDD
CRSwNP	Overall	0.91	NDD	0.85	NDD
	Consultant 1	0.89	NDD	0.85	NDD
	Consultant 2	0.91	NDD	N/A	Identical
	Consultant 3	0.91	NDD	N/A	Identical
	Consultant 4	0.89	NDD	N/A	Identical
DNS	Overall	0.53	NDD	0.07	NDD
	Consultant 1	0.76	NDD	>0.99	NDD
	Consultant 2	0.76	NDD	0.004	Visual
	Consultant 3	0.78	NDD	0.07	NDD
	Consultant 4	>0.99	NDD	0.85	NDD
Controls	Overall	0.57	NDD	>0.99	NDD
	Consultant 1	0.94	NDD	>0.99	NDD
	Consultant 2	0.94	NDD	0.15	NDD
	Consultant 3	0.94	NDD	0.41	NDD
	Consultant 4	0.57	NDD	0.27	NDD

Abbreviations: AR, allergic rhinitis; CRSsNP, chronic rhinosinusitis without nasal polyps; CRSwNP, chronic rhinosinusitis with nasal polyps; DNS, deviated nasal septum.

The best method is indicated for each comparison. N/A indicates identical results between the two methods. NDD indicates no detectable statistical difference between the two methods ( *p* > 0.05). Identical indicates both methods gave the same predictions.

In summary, RB-ES demonstrated equal sensitivity and specificity to visual inspection by ENT specialists and aligned well in most cases. Where sensitivity remained below 65%, neither method was sufficient, reinforcing the need for complementary diagnostics. While RB-ES shows promise as an adjunctive tool, most comparisons showed no detectable statistical difference. Given the study's limited power, results should be interpreted cautiously, as a lack of significance does not confirm equivalence.

### Interrater Agreements among Four ENT Specialists

Table 6 presents interrater agreement among four ENT specialists, assessed using Krippendorff's α for different classification systems. Agreement was poor for INV (α = 0.562, 95% CI: 0.504–0.613), IT (α = 0.500, 95% CI: 0.427–0.561), and DNS (α = 0.471, 95% CI: 0.404–0.532), indicating substantial variability in grading these nasal conditions. However, polyp grading demonstrated satisfactory agreement (α = 0.863, 95% CI: 0.800–0.916), suggesting it is sufficiently reliable for use as ground truth in training ML models.

**Table 6:** Interrater agreements of nasal obstruction classification and predicted diagnoses among four ENT specialists

Category	Κ-Alpha (95% CI)	Agreement level
Classifications		
INV	0.562 (0.504–0.613)	Poor agreement
IT	0.500 (0.427–0.561)	Poor agreement
DNS	0.471 (0.404–0.532)	Poor agreement
Nasal polyps	0.863 (0.800–0.916)	Satisfactory agreement
Conditions		
AR	0.291 (0.152–0.412)	Poor agreement
CRSsNP	0.127 (−0.044–0.301)	Poor agreement
CRSwNP	0.864 (0.722–0.952)	Satisfactory agreement
DNS	0.477 (0.335–0.621)	Poor agreement
Controls	0.439 (0.241–0.608)	Poor agreement

Abbreviations: AR, allergic rhinitis; CRSsNP, chronic rhinosinusitis without nasal polyps; CRSwNP, chronic rhinosinusitis with nasal polyps; DNS, deviated nasal septum; INV, internal nasal valve; IT, inferior turbinate.

95% confidence intervals were calculated using Bootstrap Method with 1,000 iterations.

For diagnosing nasal obstruction conditions, agreement was poor for AR (α = 0.291, 95% CI: 0.152–0.412), CRSsNP (α = 0.127, 95% CI: −0.044–0.301), DNS (α = 0.477, 95% CI: 0.335–0.621), and controls (α = 0.439, 95% CI: 0.241–0.608), reflecting the inconsistencies in grading INV, IT, and DNS, which are used for differential diagnosis. These findings highlight the challenges of using nasoendoscopy videos alone for diagnosing nasal obstruction. In contrast, CRSwNP demonstrated satisfactory agreement (α = 0.864, 95% CI: 0.722–0.952), indicating it is the most straightforward condition to diagnose via nasoendoscopy.

## Discussion

This pilot study compared an RB-ES to visual inspection by ENT specialists for diagnosing nasal obstruction. The RB-ES performed comparably to specialists in identifying DNS, CRSwNP, CRSsNP, and AR. However, unlike real-world nasoendoscopy, which allows clinicians to adjust angles and consider patient history, RB-ES relies solely on standardized video clips. While it provides reproducible assessments, it should be an adjunct rather than a standalone diagnostic tool. A minimum diagnostic dataset should also include allergy testing and imaging, as well as a minimum dataset of clinical symptoms, descriptors, and risk factors.

### Diagnosing Performance by Condition

- **Controls** : Visual inspection had low sensitivity (33.3%, 95% CI: 9.2–69.5%), while RB-ES improved this to 50.0% (95% CI: 18.8–81.2%), suggesting it could reduce misclassification, especially in primary care. Clinicians may overdiagnose pathology due to routine exposure to symptomatic cases. Refining endoscopic protocols or incorporating basic clinical data could mitigate this bias.
- **AR** : Both methods struggled to diagnose AR (sensitivities <65%). This is consistent with Eren et al., 30 who highlighted significant variability in the endoscopic evaluation of turbinate hypertrophy and coloration, and Koskinen et al., 31 who noted that nasoendoscopy has limited value in distinguishing AR from CRSsNP. While a ≥35% bilateral turbinate reduction postdecongestant suggests AR, chronic cases exhibit a blunted response, similar to CRSsNP. Confirmatory testing (e.g., skin prick, IgE) remains essential, and RB-ES can only support preliminary screening.
- **CRSsNP** : Diagnosing CRSsNP was challenging, with both methods showing sensitivities below 65%. This is consistent with Koskinen et al., 31 who reported limited diagnostic utility of endoscopy in differentiating CRSsNP from AR, and Cohen-Kerem et al., 32 who noted poor correlation between endoscopic findings and Lund–Mackay CT scores for CRSsNP. CT imaging and symptom-based assessments remain the gold standard, reinforcing that RB-ES should be a complementary tool.
- **CRSwNP** : RB-ES and visual inspection performed similarly ( *p* > 0.05), supporting prior evidence that nasal polyps are easily identified endoscopically. However, RB-ES does not assess deeper or posterior polyp sites, necessitating imaging when suspicion remains. Visual inspection showed slightly higher specificity for one specialist (Consultant 1), but larger studies are needed for confirmation.
- **DNS** : Both methods had borderline sensitivity (∼65%). While some specialists achieved better specificity with visual inspection, subtle nasal septal deviations remain difficult to classify endoscopically. Imaging (e.g., CT sinuses) could improve diagnostic accuracy, and refining RB-ES grading criteria may enhance consistency.

### Enhancing the Rule-Based Expert System

RB-ES provides binary outputs based on structured clinical criteria. Thresholds for turbinate hypertrophy, polyps, and DNS were pragmatically adjusted using pilot data but require validation in larger cohorts. Future iterations should account for left–right nasal asymmetries to improve sensitivity for non-polyp conditions.

Unlike ENT specialists, who integrate history and symptom duration into evaluations, RB-ES relies on standardized videos and structural grading. Incorporating clinical data, such as symptom duration, fluctuation, and allergy history, could improve accuracy, particularly for AR and CRSsNP.

### Integration into Clinical Pathways

Our findings suggest that RB-ES can support nasal obstruction diagnosis, particularly for CRSwNP, where it performs well. Nasoendoscopy remains the gold standard for nasal cavity assessment, 33 34 but diagnostic variability among clinicians is well-documented. 35 36 37 Incorporating RB-ES into existing workflows could streamline screening, triage, and training, particularly for less experienced practitioners. When RB-ES flags ambiguous cases, specialists could perform more detailed assessments, optimizing resource use and ensuring expert evaluation where most needed. 38 Rather than replacing specialist judgment, RB-ES enhances decision-making by reducing variability and standardizing assessments.

### Artificial Intelligence and Rule-Based Expert System

While RB-ES currently relies on rule-based logic, integrating ML and deep learning (DL) could enhance its diagnostic accuracy. AI-driven systems, such as convolutional neural networks and transformers, have proven effective in gastroenterology for detecting colorectal polyps and gastric cancer. 20 Similar applications are emerging in rhinology, with ML and DL models used to classify nasal pathologies, segment anatomical structures, 21 22 detect nasal polyps, 18 19 and differentiate nasal masses. 23 24 25 Automating the grading of INV, IT, DNS, and polyps using AI trained on expert consensus could improve reproducibility and reduce interrater variability. RB-ES's rule-based structure complements ML-driven grading by balancing transparency with adaptability. By combining structured clinical logic with AI-driven pattern recognition, future iterations could refine diagnostic accuracy while maintaining interpretability—key for clinical adoption.

### Interrater Reliability

Significant variability was observed among specialists, especially for AR and CRSsNP. The inability to adjust angles or question patients likely contributed to discrepancies. Future studies could improve reliability by creating reference video libraries with annotated cases and establishing consensus-based grading protocols. Calibration workshops may further enhance consistency in both human and automated evaluations.

### Expanding the Minimum Diagnostic Dataset

This study's small, imbalanced cohort ( *n* = 71) limited overall accuracy reporting. With only 43% power, the lack of statistical significance ( *p* > 0.05) does not imply equivalence between methods. A sample size of approximately 175 was required for 80% power, underscoring the need for larger, more balanced cohorts.

Beyond endoscopic grading, a comprehensive diagnostic dataset should capture key symptoms (nasal blockage, discharge, olfactory dysfunction, facial pain, epistaxis, allergy history), duration (persistent vs. intermittent), and laterality (unilateral vs. bilateral) of the nasal symptoms. A multimodal diagnostic model integrating structured clinical data with automated endoscopic analysis holds the greatest promise for accurately diagnosing nasal obstruction.

## Conclusion

This study introduces the first RB-ES for diagnosing multiple nasal obstruction conditions from nasoendoscopy videos. Despite a limited sample size and low power, results suggest the RB-ES approximates ENT specialists' judgments, particularly for CRSwNP. However, for AR, CRSsNP, DNS, and controls, both RB-ES and visual inspection alone are insufficient, underscoring the need for complementary tests like allergy testing or CT scans. By encoding clinical expertise into a transparent, rule-based system, RB-ES can standardize assessments and reduce variability, but should serve as an adjunct, not a standalone tool, due to potential missed pathologies. Future AI integration could automate and refine this framework, improving triage, diagnostic consistency, and clinical decision-making, pending validation in larger, more diverse populations.
